# Estimating the magnitude of female genital mutilation/cutting in Norway: an extrapolation model

**DOI:** 10.1186/s12889-016-2794-6

**Published:** 2016-02-02

**Authors:** Mai M. Ziyada, Marthe Norberg-Schulz, R. Elise B. Johansen

**Affiliations:** 1 Norwegian Centre for Violence and Traumatic Stress Studies, P.b. 181 Nydalen, 0409 Oslo, Norway; 2 Samfunnsøkonomisk analyse (Formerly DAMVAD Norge AS), Olavsvei 112, 1450 Nesoddtangen, Norway

**Keywords:** Female genital mutilation/cutting, Female circumcision, Prevalence, Risk, Diaspora, Norway

## Abstract

**Background:**

With emphasis on policy implications, the main objective of this study was to estimate the numbers of two main groups affected by FGM/C in Norway: 1) those already subjected to FGM/C and therefore potentially in need for health care and 2) those at risk of FGM/C and consequently the target of preventive and protective measures. Special attention has been paid to type III as it is associated with more severe complications.

**Methods:**

Register data from Statistics Norway (SSB) was combined with population-based survey data on FGM/C in the women/girls’ countries of origin.

**Results:**

As of January 1^st^ 2013, there were 44,467 first and second-generation female immigrants residing in Norway whose country of origin is one of the 29 countries where FGM/C is well documented. About 40 pct. of these women and girls are estimated to have already been subjected to FGM/C prior to immigration to Norway. Type III is estimated in around 50 pct. of those already subjected to FGM/C. Further, a total of 15,500 girls are identified as potentially at risk, out of which an approximate number of girls ranging between 3000 and 7900 are estimated to be at risk of FGM/C.

**Conclusion:**

Reliable estimates on FGM/C are important for evidence-based policies. The study findings indicate that about 17,300 women and girls in Norway can be in need of health care, in particular the 9100 who are estimated to have type III. Preventive and protective measures are also needed to protect girls at risk (3000 to 7900) from being subjected to FGM/C. Nevertheless, as there are no appropriate tools at the moment that can single these girls out of all who are potentially at risk, all girls in the potentially at risk group (15,500) should be targeted with preventive measures.

## Background

The World Health Organization (WHO) defines Female Genital Mutilation/Cutting (FGM/C) as ‘all procedures that involve partial or total removal of the external female genitalia, or other injury to the female genital organs for non-medical reasons’ [1]. FGM/C is associated with series of immediate and long-term physical and psychological health consequences [2–7].

It is estimated that 133,000,000 girls and women in 29 countries have undergone FGM/C, and that 3,600,000 girls are at risk annually [8]. Immigration from these countries to other parts of the world has made FGM/C a global concern. As a response, many governments in host countries have established preventive, protective and prosecutive measures alongside health care provision to tackle the issue [9–21]. The target group for preventive and protective measures is girls at risk, while health care provision targets girls and women already subjected to the procedure. Efficient planning and allocation of resources for the different sets of measures require precise estimates of total numbers of women and girls in each of the two target groups.

Currently, the most accurate estimates on the prevalence of FGM/C and those at risk are derived from population-based survey data such as Demographic Health Survey (DHS) and Multiple Indicator Cluster Survey (MICS) in 29 countries where FGM/C is traditionally practiced [22]. To employ similar methods in diaspora, where FGM/C is only common among immigrant minorities, would be methodologically, ethically and financially difficult. Therefore, alternative models and data-sources have been explored. The most commonly used alternative model in Europe extrapolates prevalence data from countries of origin to the corresponding resident female immigrant population [23]. Other models include surveys among health professionals [23–25] and non-representative samples of FGM/C practicing communities [23, 26, 27]. Even though the results from these surveys cannot be generalized, it still provides important insight into how FGM/C is evolving in the diaspora.

In Norway, a previous risk estimate was published in 2008 by the Norwegian Institute for Social Research (ISF) [28]. The estimate was a small part of a larger study that focused on incidence of FGM/C and included only women and girls between 0 and 19 years of age from six African countries [28].

To give a more comprehensive estimate on the number of women and girls in Norway living with FGM/C and girls at risk of FGM/C we included in this study both first and second generation immigrants from the 29 FGM/C prevalent countries. An earlier and less refined version of this study was published by DAMVAD and NKVTS in 2014 [29]. The earlier version did not take into account differences in prevalence when estimating the number of girls at risk for FGM/C; neither did it include data on typology. The present paper controls for both these factors, as well as employing updated national prevalence data.

## Methods

We adopted the extrapolation model to estimate the number of girls and women living in Norway by January 1^st^ 2013 who were at risk of FGM/C and those who most likely already have been subjected to FGM/C. We combined data on FGM/C from the 29 FGM/C prevalent countries with register data on first- and second-generation female immigrants from these countries.

### Methodological approaches

In 2012, a consortium of researchers were commissioned by the European Institute for Gender Equality (EIGE) to map the current situation on FGM/C in the European Union (EU) and Croatia [30]. The study identified lack of harmonized approach to generate reliable data on the magnitude of FGM/C as one of the main challenges facing the development of effective policies on FGM/C in the EU [23, 30]. The study also found that even when the extrapolation model was adopted, the variation in definitions and data sources have generated incomparable data [23, 30].

To enhance the comparability of our findings with other study findings in the region, we have therefore adhered as close as possible to the definitions, models and methodological approaches recommended by the aforementioned consortium of researchers in their 2103 [30] and 2015 [31] reports, as well as other key articles [23, 32, 33].

The extrapolation model combines prevalence data from countries of origin with data on resident population in the diaspora with origins from these countries [23]. So far national prevalence rates have been predominantly used in prevalence studies using this model [23] with the exception of a Dutch study [33]. One of the limitations of using national prevalence rates is that it obscures the variation in prevalence levels among different ethnicities and/or regions in the different countries [22, 23, 33]. Not adjusting for ethnicity could lead to bias in form of under- or over- estimation. In most European countries, including Norway, the National Registry offices do not provide information on ethnicity for ethical and legal reasons [23]. Nevertheless, as there is a close correlation between ethnicities and sub-regions within countries of origin [22], adjusting for regional differences could be a viable alternative [23]. A study carried out in the Netherland has therefore used data on places of birth to regroup the female immigrants according to regions within their countries of origin [33]. In Norway, additional data on place of birth could be requested from Statistics Norway. Unfortunately, since this recommendation was first published after we had requested and acquired our dataset, we were unable to adjust for neither ethnicity nor regional differences. This could therefore lead to a possible bias in our results in the form of under- or over- estimation. This bias could still be insignificant for countries with small numbers of immigrants and countries of high national prevalence. However, for countries with low national prevalence but larger immigrant groups such bias is more likely to affect the overall estimate. In this study Iraq is such an example. The national prevalence of FGM/C in Iraq is 8 pct. whereas it is as high as 42.8 pct. among the Kurdish population. According to estimates from the Norwegian office of immigration (UDI), a little over 40 pct. of the Iraqi residents in Norway are of Kurdish origin. If this factor is taken into account, the total number of Iraqis affected by FGM/C in Norway will be much higher than our current estimates.

Another limitation of the extrapolation of national prevalence level is that it does not address the selection process of immigrants [32]. Ortensi et al. [32] argue that migration is a selective process and that immigrants are usually younger, wealthier, and more educated than their counterpart that did not migrate. Since younger age and higher levels of wealth and education often are correlated with lower prevalence levels of FGM/C, the authors emphasize that the application of the national prevalence levels without adjustment for age, wealth and education is likely to bias the indirect estimates of FGM/C prevalence [32]. Again, it was unfortunate that this recommendation was published after finalization of the data analysis and subsequent deletion of dataset (stratified by age) as stipulated by Statistics Norway. The suggested improved model will nevertheless be incorporated in any future follow-up studies.

### Prevalence estimation

Leye et al. [23] defined the prevalence of FGM/C in any EU member state as ‘*the number of women and girls in that country who have undergone FGM at a certain point in time expressed as the proportion of the total number of women living in the country, but originating from countries where FGM is practiced*’.

In this article, we adopted a similar definition for the prevalence of FGM/C in Norway: The proportion of girls and women estimated to have undergone FGM/C at a certain point in time out of the total number of women and girls with origins from the 29 FGM/C prevalent countries.

Identifying the relevant populations and sub-populations is central to both prevalence and risk estimation of FGM/C. The total resident population originating from the 29 FGM/C prevalent countries in Norway consists of first- and second-generation immigrants. In this article, we use the term first- generation immigrants to refer to girls and women who migrated from one of the 29 countries where FGM/C is concentrated, whereas second- generation immigrants refers to girls born in Norway to two parents who have migrated from one of these 29 countries. Girls with only one parent from a FGM/C practicing country were excluded, as the risk is very uncertain and most likely low.

The first-generation immigrants consist of those who have been subjected to FGM/C prior to arrival in host countries, those who have been subjected to FGM/C post arrival, those who have not been subjected to FGM/C because they/their families do not practice or have abandoned the practice, and those who have not been subjected to FGM/C but still are at risk.

The second-generation group consists of those who have been subjected to FGM/C at one point of time, those who have not been subjected to FGM/C and are not at risk as their families do not practice or have abandoned the practice and those who have not been subjected to FGM/C but still at risk.

Thus the relevant groups for estimation of FGM/C prevalence would be:

1. First-generation immigrants who have been subjected to FGM/C prior to arrival in host countries and those who have been subjected to FGM/C post arrival.
2. Second-generation immigrants who have been subjected to FGM/C at one point of time.

In countries of origin, there is usually a customary age within which FGM/C is performed [22]. Thus, it would be safe to assume that those of first-generation immigrants who were older than that age upon arrival in host countries, had already been subjected to FGM/C in corresponding proportion to the prevalence rates in their countries of origin.

The challenge would be to estimate the number of second-generation immigrants who have been subjected to FGM/C, as well as those of the first-generation immigrants who have been subjected to FGM/C post migration (those who were younger than the customary age upon arrival but older than that age by the time of analysis/estimation). This challenge arises from the lack of reliable FGM/C incidence rates in host countries and the expected overestimation if extrapolation of prevalence rates from countries of origin were to be used. There is growing evidence on attitude change toward FGM/C in host countries that would indicate FGM/C is practiced at much lower rates than in countries of origin [34–37]. Therefore, we have decided to assume that none of the first-generation immigrants who were younger than the customary age of FGM/C in their countries of origin upon arrival in Norway or the second-generation immigrants has been subjected to FGM/C.

### FGM/C risk estimation

The 2015 EIGE report [31] emphasized the difference between *girls potentially* at risk and *girls at risk*.

Girls potentially at risk are defined as ‘*minor girls (in the age range of 0–18) who come from FGM risk countries, or were born to parents (or one parent) who originate from countries where female genital mutilation is commonly practiced*’ [31]. We found it necessary to modify this definition as including all first-generation girls under the age of 18 would imply including girls who could already have been subjected to FGM/C prior to migration. As a consequence we considered only first-generation girls who were younger upon arrival in Norway than the customary age of FGM/C in their countries of origin to be potentially at risk; whereas for second-generation girls, all those under 18 were considered to be potentially at risk. Further for the second-generation girls, only those with BOTH parents originating from one of the 29 countries were included. Therefore it is more apt to say that our definition for girls potentially at risk is: ‘first-generation girls who were younger upon arrival in Norway than the customary age for FGM/C in their countries of origin and second-generation girls who at the time of data collection (reference year/date) were younger than 18’. Preventive measures should target all girls potentially at risk.

On the other hand, girls at risk is not defined per se in the 2015 EIGE report [31]. Instead the report provides a definition for the FGM/C risk estimation as:

‘*minor girls (either born in, or born to mothers from, FGM risk countries) living in an EU Member State who might actually be at risk of female genital mutilation, expressed as a proportion of the total number of girls living in an EU country who originate from or are born to a mother from FGM risk countries*’ [31].

The corresponding recommended model presents the risk estimation as an interval between low and high risk scenarios that takes into consideration the effect of migration through ‘migration and acculturation impact factor’ [31]. In this model the national FGM/C prevalence rate for the age cohort 15–19 is multiplied by the total number of first and second generation immigrant girls who are younger than the customary age for cutting at their/their parents countries of origin. Here, unlike the estimation of girls potentially at risk, the customary age for cutting is taken into consideration. We do agree that the customary age for FGM/C in the countries of origin is important to exclude first-generation girls who could have been subjected to FGM/C prior to migration. However, we are skeptical to the exclusion of first-generation girls who have been younger than the customary age in their countries of origin upon arrival but older than that age at the time of data collection/reference year. The same applies to second-generation girls who were older than the customary age. This skepticism stems from the emerging evidence that customary age for cutting in countries of origin has less relevance in the migration context, where the ‘opportunity to cut’ seems to be more significant [31]. Consequently, we have only excluded first-generation girls who were older than the customary age upon arrival, first-generation girls who were younger than the customary age upon arrival but older than 18 at the reference year, and second generation girls who were also older than 18 at the reference year.

Other than this modification on the exclusion criteria, we have followed the rest of the steps described in the recommended model.

The recommended model expresses the migration and acculturating impact factor as binary variable with values as either ‘0’ or ‘1’ and is assessed through qualitative research. The ‘0’ value signifies that migration has no impact on attitudes and behaviors toward FGM/C in the migration context. That is to say that the risk of FGM/C would be the same as if migration never took place. The ‘1’ value signifies on the other hand that migration has such an impact on attitudes and behaviors toward FGM/C that the level of risk is reduced to zero.

In the high FGM/C risk scenario, it is assumed that migration has no effect whatsoever (migration and acculturation impact factor equal ‘0’) and that the number of first and second generation girls at risk of FGM/C would be the same as if they/their parents have never migrated [31].

In the low FGM/C risk scenario, it is assumed that migration has an impact on attitudes and behaviors toward FGM/C and that second-generation girls experience a lower risk. In contrast, the first-generation girls are considered to be at risk (migration and acculturation impact factor equal ‘0’). Even though for purposes of calculation the migration and acculturation impact factor for second-generation girls in this scenario is given the value ‘1’, the authors caution against interpreting this estimation in the strictest sense that no second-generation girl would be at risk [31].

The resulting estimates of the low and high risk scenarios are then presented as an interval; in which the estimates from the two scenarios demarcate its boundaries. The scenario that is more likely to apply in the local context is finally selected according to the findings from the qualitative component [31]. We have not carried out a qualitative study as recommended to accompany our estimation analysis. Still, there are several recent studies on change of attitude and behavior among Somalis in Norway [34–36] that helped to guide us as to which of the two scenarios is more relevant.

Finally, it is important to mention that despite the great potential of this model, the binary nature of the migration and acculturation impact factor is a significant limitation. To assume that migration would have either ‘no impact’ or ‘a huge impact’ on attitudes and behaviors towards FGM/C is in contrast to findings from several studies that indicate much more fluid opinions and variations in the depth or intensity of conviction [22, 38–41]. The migration and acculturation impact factor can be significantly improved by future research that can help to assign values between ‘0’ and ‘1’ dependent on scores given to identified determinants for change.

### Estimation of type III

We extrapolated the prevalence of the different types of FGM/C in countries of origin to the different groups to estimate the number of those expected to have already been subjected to the most severe form of FGM/C (infibulation) and those at risk of infibulation.

### Data sources

In this study, we used two main data sources: 1) DHS and MICS and 2) Statistics Norway (SSB).

DHS and MICS are broad population and health surveys using a statistically representative sample of the population. In both DHS and MICS surveys, the percentage of girls and women aged 15 to 49 reporting to have experienced any form of FGM/C is used to indicate how prevalent the practice is in a particular country [22]. Great consistency of data over time, as well as comparability to other studies, suggest that data on national prevalence is reliable [22, 42]. Still, it is important to acknowledge some of the inherent limitations of the DHS and MICS surveys such as reporting bias and the inability to reflect recent FGM/C trends in practicing countries [26, 32].

The DHS and MICS data used in this article are openly available and are published online in form of country reports on the respective web-sites for the responsible organizations. We used the most recent DHS and MICS reports for each of these 29 countries [43, 44] to ensure that we have the latest data on prevalence, typology and customary age for FGM/C (Tables 1 and 2). In two countries, Egypt and Sudan, the latest reports (DHS 2008 and MICS 2010 respectively) did not provide information on typologies. For these two countries we instead used data from the latest available report that has information on typology, e.g. for Egypt we used the DHS 1995 instead of DHS 2008.

**Table 1:** DHS and MICS data: prevalence levels and customary age for FGM/C

Country	Prevalence 15–49	Prevalence 15–19	Customary age for FGM/C	Data source
Benin	7,3 %	2,0 %	0–14	DHS 2011–12
Burkina Faso	75,8 %	57,7 %	0–4	DHS 2010
Cameroon	1,4 %	0,4 %	5–9	DHS 2004
Central African Republic	24,2 %	17,9 %	5–14	MICS 2010
Chad	44,2 %	41,0 %	0–9	MICS 2010
Côte d’Ivoire	38,2 %	31,3 %	0–5	DHS 2012
Djibouti	93,1 %	89,5 %	5–9	MICS 2006
Egypt	97,0 %	98,1 %	10–14	DHS 1995
Eritrea	88,7 %	78,4 %	0–2	DHS 2002
Ethiopia	74,3 %	62,1 %	0–2	DHS 2005
Gambia	76,3 %	77,1 %	0–4	MICS 2010
Ghana	3,8 %	1,5 %	4–14	MICS 2011
Guinea	96,9 %	94,0 %	0–9	MICS 2012
Guinea–Bissau	49,8 %	48,4 %	0–9	MICS 2010
Iraq	8,1 %	4,9 %	4–10	MICS 2011
Kenya	27,1 %	14,6 %	5–14	DHS 2008–09
Liberia	58,3 %	35,9 %	10–15	DHS 2007
Mali	91,4 %	90,3 %	0–4	DHS 2012–13
Mauritania	69,4 %	65,9 %	0–4	MICS 2011
Niger	2,0 %	1,4 %	0–3	DHS 2012
Nigeria	24,8 %	15,3 %	0–3	DHS 2013
Senegal	25,7 %	24,0 %	0–4	DHS 2010–11
Sierra Leone	89,6 %	74,3 %	8–18	DHS 2013
Somalia	97,9 %	96,7 %	5–9	MICS 2006
Sudan	89,2 %	86,8 %	6–8	DHS 1989–90
Togo	3,9 %	1,1 %	4–14	MICS 2010
Uganda	1,4 %	1,0 %	8–18	DHS 2011
United Republic of Tanzania	14,6 %	7,1 %	0–5	DHS 2010
Yemen	22,6 %	19,3 %	0–1	DHS 1997

**Table 2:** DHS and MICS data: prevalence of different types of FGM/C

Country	Prevalence level 15–49		Prevalence level 15–19		Data source
	Type 3	Other types or undetermined	Type 3	Other types or undetermined	
Benin	12,5 %	87,5 %	8,7 %	91,3 %	DHS 2011–12
Burkina Faso	1,2 %	98,8 %	0,7 %	99,3 %	DHS 2010
Cameroon	5 %	95 %	5 %	95 %	DHS 2004
Central African Republic	7 %	93 %	9,5 %	90,5 %	MICS 2010
Chad	7,2 %	92,8 %	8,3 %	91,7 %	MICS 2010
Côte d’Ivoire	8,7 %	91,3 %	10,4 %	89,6 %	DHS 2011–12
Djibouti	67,2 %	32,8 %	42,4 %	57,6 %	MICS 2006
Egypt	0,7 %	99,3 %	2,3 %	97,7 %	DHS 1995
Eritrea	38,6 %	61,4 %	33,8 %	66,2 %	DHS 2002
Ethiopia	6,1 %	93,9 %	4,7 %	95,3 %	DHS 2005
Gambia	8,9 %	91,1 %	6,6 %	93,4 %	MICS 2010
Ghana	8 %	92 %	6,7 %	93,3 %	MICS 2011
Guinea	7,5 %	92,5 %	7,7 %	92,3 %	MICS 2012
Guinea-Bissau	11,8 %	88,2 %	11,4 %	88,6 %	MICS 2010
Iraq	0 %	100 %	0 %	100 %	MICS 2011
Kenya	13,4 %	86,6 %	17,6 %	82,4 %	DHS 2008–09
Liberia	0 %	100 %	0 %	100 %	DHS 2007
Mali	10,6 %	89,4 %	11,1 %	88,9 %	DHS 2012–13
Mauritania	0 %	100 %	0 %	100 %	MICS 2011
Niger	6,3 %	93,7 %	8,0 %	92,0 %	DHS 2012
Nigeria	5,3 %	94,7 %	3,6 %	96,4 %	DHS 2013
Senegal	13,8 %	86,2 %	10,9 %	89,1 %	DHS 2010–11
Sierra Leone	9,0 %	91,0 %	10,1 %	89,9 %	DHS 2013
Somalia	79,3 %	20,7 %	76,0 %	24,0 %	MICS 2006
Sudan	82,3 %	17,7 %	73,9 %	26,1 %	DHS 1989–90
Togo	5,1 %	94,9 %	0 %	100 %	MICS 2010
Uganda	0 %	100 %	0 %	100 %	DHS 2011
United Republic of Tanzania	0,7 %	99,3 %	0,8 %	99,2 %	DHS 2010
Yemen	0 %	100 %	0 %	100 %	DHS 1997

Data on the most extensive form of FGM/C, type III, (Table 2) was available for most of the countries with a large immigrant group in Norway. However, typology data are missing for Liberia, Iraq, Uganda and Yemen. Therefore we assumed a zero prevalence of type III in these four countries. Generally, available data on types of FGM/C is found to be much less reliable than prevalence data, with a tendency to under-report type III [45, 46].

The dataset for this study was requested and obtained from our second data source i.e. Statistics Norway (SSB) which provides micro-data for research projects. Using this dataset required exemption from the duty of confidentiality and was further handled with utter adherence to SSB ethical requirements. The dataset is population register data that covers all female residents in Norway that, by the reference date (January 1^st^ 2013), have migrated or their parents have migrated from one of the aforementioned 29 countries (Table 3). This includes refugees, whereas women and girls visiting Norway for less than six months, asylum seekers and illegal immigrants are not included. The dataset includes information on countries of origin, age at arrival in Norway and current age. It also comprises information on parents’ countries of origin for second-generation immigrants.

**Table 3:** Statistic Norway dataset on first and second generation immigrants from the 29 countries by groups

Country	Group 1			Group 2		Total
	Group 1a	Group 1b	Group 1c	Group 2a	Group 2b	
Cameroon	204	11	^a^	34	0	257
Côte d’Ivoire	99	11	^a^	15	^a^	127
Djibouti	30	^a^	^a^	10	^a^	46
Egypt	203	30	42	44	^a^	326
Eritrea	4508	218	10	606	107	5449
Ethiopia	2707	143	^a^	528	52	3434
Gambia	432	21	^a^	151	47	654
Ghana	582	133	47	226	62	1050
Guinea	73	29	^a^	17	^a^	127
Iraq	7285	1949	416	3422	146	13218
Kenya	643	120	64	50	17	894
Liberia	310	100	40	74	^a^	526
Nigeria	529	42	^a^	142	27	749
Senegal	71	^a^	^a^	22	^a^	102
Sierra Leone	138	54	37	49	10	288
Somalia	8873	1630	593	3654	206	14956
Sudan	640	78	116	134	^a^	977
Togo	38	^a^	^a^	14	0	62
Uganda	311	124	44	39	16	534
United Republic of Tanzania	340	42	8	36	^a^	432
Yemen	135	^a^	0	38	^a^	186
Other countries^b^	51	^a^	^a^	19	^a^	73
*Total*	*28202*	*4763*	*1456*	9324	722	44467
%	63 %	11 %	3 %	21 %	2 %	100 %

^a^More than 0 and less than 10 per category

^b^Countries with less than 20 in total: Benin, Burkina Faso, Central African Republic, Chad, Guinea-Bissau, Mali, Mauritania and Niger

### Data analysis

As of 1^st^ January 2013 there were 44,467 girls and women who have either immigrated or their parents have immigrated from one of the 29 FGM/C prevalent countries (Table 3). We first divided these 44,467 girls and women into two main groups: 1) first-generation immigrants and group 2) second-generation immigrants. We further divided the first group into three groups (1a, 1b and 1c) taking into account the following variables: customary age for FGM/C in the country of origin, age at arrival in Norway and current age.

Group 1a consisted of 28,202 girls and women who upon arrival in Norway were older than the customary age for FGM/C in their countries of origin (Table 3). Hence, a corresponding proportion to the 15–49 prevalence rates in countries of origin is most likely to have already been subjected to the practice.

Group 1b consisted of 4763 girls who upon arrival in Norway were younger than or within the customary age for FGM/C in their countries of origin, and who by 1^st^ January 2013 were older than the customary age but younger than 18. Group 1c, consisted of 1456 girls who upon arrival in Norway were younger than or within the customary age for FGM/C in their countries of origin, and who were still within the customary age or younger by January 1^st^ 2013. Both groups were assumed not to have been subjected to FGM/C. Instead, these two groups were considered to be potentially at risk, and that a proportion of this group corresponding to that of girls in countries of origin in the age group 15–19, are at risk of FGM/C.

Group 2, second-generation girls constituted 10,046 girls of which 9324 (group 2a) were under the age of 18 by January 1^st^ 2013. We assumed that girls under 18 with both parents originally from one of the 29 countries (9324) were potentially at risk and that a proportion of this group corresponding to that of girls in countries of origin in the age group 15–19 are at risk of FGM/C. In limited cases where parents were from two different FGM/C practicing countries, subsequent estimation was based on prevalence levels from the mother’s country of birth. Girls with only one parent from FGM/C practicing country were excluded, as the risk is very uncertain and most likely low.

To calculate the total number of girls potentially at risk, we combined group 1b, 1c and 2a without adjusting for prevalence rates in their parents’ countries of origin. To estimate the FGM/C risk we adopted the EIGE model described earlier in this section.

Finally, to estimate the number of those who have already been infibulated and the number of girls at risk of infibulation, we extrapolated the prevalence of the different types of FGM/C as reported in DHS and MICS (Table 2) to the different groups.

## Results

In this study we have divided girls and women into three major categories: 1) first- and second- generation girls and women who most likely have been subjected to FGM/C prior to immigration, 2) first- and second- generation girls who are potentially at risk of FGM/C, and 3) first- and second- generation girls that are at risk of FGM/C.

**Fig. 1:**
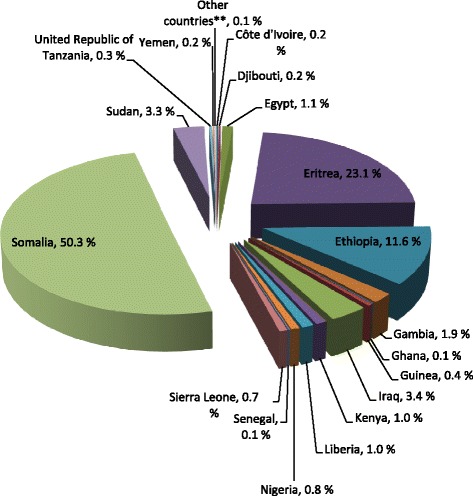
FGM/C Percentage of girls and women already subjected to FGM/C in Norway by country of origin

**Fig. 2:**
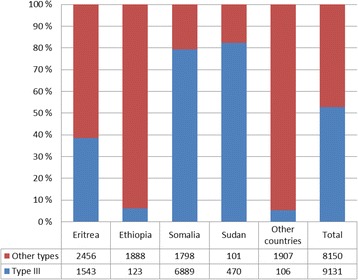
Numbers of girls and women subjected to FGM/C type III by most represented countries of origin

As of January 1^st^ 2013, around 17,300 girls and women are estimated to have already been subjected to FGM/C prior to immigration to Norway, constituting an approximate prevalence of 40 pct. (Table 4). 50 pct. of these 17,300 girls and women originate from Somalia (Fig. 1). Also, around 50 pct. of the 17,300 girls and women are estimated to have been infibulated (Table 5). The majority of those estimated to be infibulated originate from Somalia, Eritrea, Sudan and Ethiopia (Fig. 2). These numbers nevertheless need to be interpreted with caution as data on types are significantly less reliable than prevalence data.

**Table 4:** Estimated numbers of girls and women already undergone FGM/C

Country	Undergone FGM/C^a^ (Group 1a)
Cameroon	^b^
Côte d’Ivoire	38
Djibouti	28
Egypt	197
Eritrea	3999
Ethiopia	2011
Gambia	330
Ghana	22
Guinea	71
Iraq	590
Kenya	174
Liberia	181
Nigeria	131
Senegal	18
Sierra Leone	124
Somalia	8687
Sudan	571
Togo	^b^
Uganda	^b^
United Republic of Tanzania	50
Yemen	31
Other countries^c^	22
*Total*	*17281*
*% of the total FGM/C affected population*	*38.9 %*

^a^Adjusted for prevalence level 15–49

^b^More than 0 and less than 10 per category

^c^Countries with less than 20 in total: Benin, Burkina Faso, Central African Republic, Chad, Guinea-Bissau, Mali, Mauritania and Niger

**Table 5:** Girls and women already subjected to FGM/C by types

Country	Already subjected to FGM/C		
	Type III	Other types	Total
Cameroon	0	^a^	^a^
Côte d’Ivoire	3	35	38
Djibouti	19	9	28
Egypt	^a^	196	197
Eritrea	1543	2456	3999
Ethiopia	123	1888	2011
Gambia	30	300	330
Ghana	^a^	20	22
Guinea	^a^	66	71
Iraq	0	590	590
Kenya	23	151	174
Liberia	0	181	181
Nigeria	^a^	124	131
Senegal	^a^	15	18
Sierra Leone	11	113	124
Somalia	6889	1798	8687
Sudan	470	101	571
Togo	0	^a^	^a^
Uganda	0	^a^	^a^
United Republic of Tanzania	0	49	49
Yemen	0	31	31
Other countries^b^	^a^	19	21
Total	9131	8150	17281
*%*	*52.8 %*	*47.2 %*	*100 %*

^a^More than 0 and less than 10 per category

^b^Countries with less than 20 in total: Benin, Burkina Faso, Central African Republic, Chad, Guinea-Bissau, Mali, Mauritania and Niger

On the other hand, 15,500 girls are estimated to be potentially at risk (Table 6), while an approximate number of girls ranging between 3000 and 7900 were estimated to be at risk (Table 7). Finally, out of the total number of girls at risk, a number of girls ranging between 1800 and 4800 are estimated to be at risk of infibulation (Table 8).

**Table 6:** Girls potentially at risk

Country	Group 1		Group 2	Total
	Group 1b	Group 1c	Group 2a	
Cameroon	11	^a^	34	53
Côte d’Ivoire	11	^a^	15	27
Djibouti	^a^	^a^	10	13
Egypt	30	42	44	116
Eritrea	218	10	606	834
Ethiopia	143	^a^	528	675
Gambia	21	^a^	151	175
Ghana	133	47	226	406
Guinea	29	^a^	17	52
Iraq	1949	416	3422	5787
Kenya	120	64	50	234
Liberia	100	40	74	214
Nigeria	42	^a^	142	193
Senegal	^a^	^a^	22	30
Sierra Leone	54	37	49	140
Somalia	1630	593	3654	5877
Sudan	78	116	134	328
Togo	^a^	^a^	14	24
Uganda	124	44	39	207
United Republic of Tanzania	42	8	36	86
Yemen	^a^	0	38	46
Other countries^b^	^a^	^a^	19	26
*Total*	*4763*	*1456*	9324	15543
%	11 %	3 %	21 %	100 %

^a^More than 0 and less than 10 per category

^b^Countries with less than 20 in total: Benin, Burkina Faso, Central African Republic, Chad, Guinea-Bissau, Mali, Mauritania and Niger

**Table 7:** Estimated numbers of girls at risk of FGM/C: low and high risk scenarios

Country	At risk of FGM/C	
	Low FGM risk scenario^a^	High FGM risk scenario^b^
Cameroon	0	0
Côte d’Ivoire	^c^	^c^
Djibouti	^c^	12
Egypt	71	114
Eritrea	179	654
Ethiopia	91	419
Gambia	19	135
Ghana	^c^	^c^
Guinea	33	49
Iraq	116	284
Kenya	27	34
Liberia	50	77
Nigeria	^c^	30
Senegal	^c^	^c^
Sierra Leone	68	104
Somalia	2150	5683
Sudan	168	285
Togo	0	0
Uganda	^c^	^c^
United Republic of Tanzania	^c^	^c^
Yemen	^c^	^c^
Other countries^d^	^c^	12
*Total*	*2999*	*7929*

^a^Group 1b + 1c adjusted for prevalence level 15–19

^b^Group 1b + 1c + 2a adjusted for prevalence 15–19

^c^Countries with less than 20 in total: Benin, Burkina Faso, Central African Republic, Chad, Guinea-Bissau, Mali, Mauritania and Niger

**Table 8:** Girls at risk of different types of FGM/C

Country	Low FGM/C risk scenario		High FGM/C risk scenario	
	Type III	Other types	Type III	Other types
Cameroon	0	0	0	0
Côte d’Ivoire	0	^a^	^a^	^a^
Djibouti	^a^	^a^	^a^	^a^
Egypt	^a^	69	^a^	111
Eritrea	60	118	221	433
Ethiopia	^a^	87	20	399
Gambia	^a^	17	^a^	126
Ghana	0	^a^	0	^a^
Guinea	^a^	30	^a^	45
Iraq	0	116	0	284
Kenya	^a^	22	^a^	28
Liberia	0	50	0	77
Nigeria	0	^a^	^a^	28
Senegal	0	^a^	^a^	^a^
Sierra Leone	^a^	61	11	94
Somalia	1634	516	4319	1364
Sudan	124	44	210	74
Togo	0	0	0	0
Uganda	0	^a^	0	^a^
United Republic of Tanzania	0	^a^	0	^a^
Yemen	0	^a^	0	^a^
Other countries^b^	0	^a^	^a^	^a^
Total	1842	1157	4811	3118
*%*	*61 %*	*39 %*	*61 %*	*39 %*

^a^More than 0 and less than 10 per category

^b^Countries with less than 20 in total: Benin, Burkina Faso, Central African Republic, Chad, Guinea-Bissau, Mali, Mauritania and Niger

## Discussion

FGM/C prevalence and risk estimations are needed to guide effective policies and interventions on health care and prevention. Prevalence and risk estimates can help to adjust the allocation of resources to the actual needs, as well as to evaluate the results of various interventions.

Women and girls who have been subjected to FGM/C (estimated to be around 17,300 in this study) are in potential need of health care for related physical and psychological complications. Need for health care is expected to be particularly important for those who have undergone infibulation (approximately 9100 out of the 17,300). Prevalence estimates, together with data on service provision and utilization, could also be useful in the assessment of accessibility and acceptability of available services.

Risk estimates are necessary to guide preventive measures. Generally, the main objective of preventive measures is to prevent FGM/C among girls at risk, who we found in this study to be in the range of 3000 to 7900. Currently, there are no appropriate tools, such as prediction models, that can single out these girls from all who are potentially at risk. Therefore, all girls who are potentially at risk, 15,500 girls, are to be targeted with preventive measures. Still additional protective measures may be needed for the girls at risk. Further, when combined with incidence rates, estimates of girls at risk can be useful in the evaluation of preventive measures and assessment of change.

Prevalence and risk estimates can also help to compare the magnitude of FGM/C between different countries. Currently, there are about 16 studies from nine European countries that estimate the FGM/C prevalence and/or risk by extrapolating DHS and MICS data to their immigrant population [23, 32, 47]. However, beside Norway, only three other countries use register-based data (Italy, Netherlands and Belgium) which would lead to more accurate estimates [23, 32, 33, 48]. The rest rely on census data, that collect information on country of origin from about five percent of the citizens, to estimate their female population originating from FGM/C prevalent countries [23]. Limitation of census data in such a context is that uneven residence patterns of migrants in most countries, and other challenges such as language barriers or resistance to participation, can give inaccurate numbers of residents.

It is not possible to directly compare our prevalence estimates to that in other European countries. That is as even when the extrapolation model is used, there is a wide variation regarding who is included. In a recent study from the UK, all female 15 years of age and above emigrating from FGM/C prevalent countries were considered to have already undergone FGM/C in proportion to the 15–49 prevalence in home countries [47]. In Belgium, all immigrants, regardless of age upon arrival, are considered to have FGM/C but in proportion to the prevalence in countries of origin [48]. The closest to our approach is a recent study from the Netherlands. In the Dutch study, all immigrant females who upon arrival were above the median age for FGM/C in their home countries were considered to have already been cut, but in proportion to the corresponding age cohort [33]. In our study instead of using median age as in the Dutch study, we used the upper margin of the customary age as a cut-off point.

Comparing estimates of girls at risk is even more complex. Even though only few European countries have estimates on FGM/C risk so far, the variation in the definition of who is at risk is vast. Second-generation is considered to be at risk in proportion to the prevalence in parents’ country of origin in Belgium [48]. In UK, it is the first-generation under 15 that is considered as at risk [47]. In the Netherlands, there are three alternative estimates for girls at risk. Two out of the three alternatives consider the second-generation to be at no risk [33].

In addition, in this study we decided to include only girls whose both parents are from countries with a tradition of FGM/C. In contrast, most other studies include all girls, even those with only one parent from FGM/C prevalent countries [33, 47, 48]. We believe that our approach gives more accurate estimates, as the risk is expected to be significantly lower when one of the parents is from a non-practicing country.

Nevertheless, the results from the three recent studies from Ireland, Sweden and Portugal that have piloted the recommended model for risk estimation presented in the 2015 EIGE report [31] could, to some degree, be comparable to our findings. In Ireland, the girls potentially at risk in 2012 were 14,577, of which 70 pct. originated from Nigeria (a low prevalent country). Of these girls the number estimated to be at risk varied between 158 and 1632. In Norway, the estimates for girls potentially at risk in 2013 were very close to the estimates from Ireland (15,500). Nevertheless in the high risk scenario we can see a substantial difference between those expected to be at risk in Ireland (1632) and their counterpart in Norway (7900). A similar difference can also be observed between our study and those from Sweden and Portugal. In Sweden, 59,409 girls were potentially at risk in 2011. Of these girls the number estimated to be at risk varied between 2016 and 11,145. This substantial difference, between our estimates and those of the EIGE model’s pilot countries, can be attributed to the difference in the composition of the potentially at risk groups in the different countries. Unlike Ireland and Portugal, a large number of girls in Norway have origins from high prevalence countries such as Somalia, Eritrea and Ethiopia. The difference can also be attributed to the different inclusion criteria for both the potentially at risk and the at risk groups in our analysis and in the EIGE model. In our analysis, unlike the EIGE model, girls under 18 who upon arrival were older than the customary age in their countries of origin were excluded from the potentially at risk. This group was only excluded in the EIGE model while estimating the numbers of girls at risk. Further, we did not exclude from our estimates of girls at risk those under 18 who were older than the customary age for FGM/C in countries of origin per the reference year. Instead, taking as a point of departure the emerging evidence that ‘opportunity to cut’ is more relevant in countries of migration [31], we included in our estimation all second-generation girls under 18, as well as first-generation girls under 18 who were younger than the customary age for FGM/C upon arrival.

The three above mentioned studies had an accompanying qualitative component to assess change in attitudes and behaviors toward FGM/C among the affected groups following immigration. All three studies reported to find change and consequently concluded that the estimates in the low FGM/C risk could be more accurate [31]. We have not conducted the recommended qualitative component. Still, there are several recent studies that have assessed attitudes and behaviors towards FGM/C among Somalis in Norway [34–36]. The reported change in these studies would indicate that similar to Ireland, Portugal and Sweden, the estimates in the low FGM/C risk scenario could be more accurate.

## Conclusion

Reliable estimates on FGM/C are important for evidence-based policy making. Thus in the absence of population based representative surveys, the extrapolation model is currently the most viable way to estimate both prevalence and risk in diaspora.

In this study we aspired to estimate the number of two main groups with different policy implications: those who are already subjected to FGM/C and those at risk.

We calculated the number of girls and women who are expected to have already undergone FGM/C. Also, as type III is associated with higher health risks and health care needs, we identified the proportion of those expected to be infibulated. Our estimate suggests that around 50 pct. of the 17,300 girls and women estimated to have undergone FGM/C prior to immigration had type III. Of the girls at risk in both the low and high risk scenario, approximately 60 pct. were at risk of infibulation.

## Abbreviations

DHS: Demographic Health Survey
EIGE: European Institute for Gender Equality
EU: European Union
FGM/C: Female Genital Mutilation/Cutting
ISF: Norwegian Institute for Social Research
MICS: Multiple Indicator Cluster Survey
NKVTS: Norwegian Center for Violence and Traumatic Stress Studies
SSB: Statistics Norway
WHO: World Health Organization
